# Exacerbations of chronic obstructive pulmonary disease: when are antibiotics indicated? A systematic review

**DOI:** 10.1186/1465-9921-8-30

**Published:** 2007-04-04

**Authors:** Milo A Puhan, Daniela Vollenweider, Tsogyal Latshang, Johann Steurer, Claudia Steurer-Stey

**Affiliations:** 1 Horten Centre, University Hospital of Zurich, Postfach Nord, CH-8091 Zurich, Switzerland

## Abstract

**Background:**

For decades, there is an unresolved debate about adequate prescription of antibiotics for patients suffering from exacerbations of chronic obstructive pulmonary disease (COPD). The aim of this systematic review was to analyse randomised controlled trials investigating the clinical benefit of antibiotics for COPD exacerbations.

**Methods:**

We conducted a systematic review of randomised, placebo-controlled trials assessing the effects of antibiotics on clinically relevant outcomes in patients with an exacerbation. We searched bibliographic databases, scrutinized reference lists and conference proceedings and asked the pharmaceutical industry for unpublished data. We used fixed-effects models to pool results. The primary outcome was treatment failure of COPD exacerbation treatment.

**Results:**

We included 13 trials (1557 patients) of moderate to good quality. For the effects of antibiotics on treatment failure there was much heterogeneity across all trials (I^2 ^= 82%). Meta-regression revealed severity of exacerbation as significant explanation for this heterogeneity (p = 0.016): Antibiotics did not reduce treatment failures in outpatients with mild to moderate exacerbations (pooled odds ratio 1.09, 95% CI 0.75–1.59, I^2 ^= 18%). Inpatients with severe exacerbations had a substantial benefit on treatment failure rates (pooled odds ratio of 0.25, 95% CI 0.16–0.39, I^2 ^= 0%; number-needed to treat of 4, 95% CI 3–5) and on mortality (pooled odds ratio of 0.20, 95% CI 0.06–0.62, I^2 ^= 0%; number-needed to treat of 14, 95% CI 12–30).

**Conclusion:**

Antibiotics effectively reduce treatment failure and mortality rates in COPD patients with severe exacerbations. For patients with mild to moderate exacerbations, antibiotics may not be generally indicated and further research is needed to guide antibiotic prescription in these patients.

## Background

The use of antibiotics in exacerbations of chronic obstructive pulmonary disease (COPD) remains controversial [1,2]. It is unclear which patients should receive antibiotics. The uncertainty arises from a complex clinical situation where the cause of the exacerbation is often unidentifiable [3]. Around 40–50% of exacerbations may be attributed to bacteria while other causes include viral infections or environmental irritants [4-6]. Even if bacteria are identified, it is uncertain whether they actually caused the exacerbation or whether they were present as part of the flora before the exacerbation.

Diagnostic tests cannot reliably distinguish between bacterial, viral or other origins of exacerbations. As a consequence, many physicians decide to be on the "safe" side and prescribe antibiotics[7]. The uncertain role of antibiotics is reflected by current guidelines that insufficiently inform physicians about adequate prescription of antibiotics [3,8]. Guidelines suggest adding an antibiotic if sputum is purulent, if sputum volume is increased and/or if fever is present. However, evidence supporting this suggestion is not based on randomised trials. There are no randomised trials where prescription of antibiotics was guided by purulence of sputum or other criteria. In addition, the extent of symptom worsening is difficult to standardise and utility of sputum assessment is controversial [9,10].

A systematic review of randomised, placebo-controlled trials could inform the debate about the role of antibiotics substantially. Eleven years ago, a meta-analysis suggested a small improvement of lung function by antibiotics in COPD patients with an exacerbation, but the review was limited by the restriction to articles in English and its focus on lung function [11]. A recent systematic review [12] considered patient-important outcomes but missed some studies and included a non-randomised trial[13]. Inclusion of all available trials is, however, crucial to avoid selection bias and to study factors modifying the effects of antibiotics such as severity of exacerbation. Therefore, our aim was to review all randomised placebo-controlled trials that assessed the effects of antibiotics on patient-important outcomes in COPD patients suffering from exacerbations.

## Methods

### Selection criteria

We included randomised controlled trials comparing any antibiotics with placebo or no antibiotics in COPD patients suffering from an acute exacerbation defined as a worsening of a previous stable situation with symptoms such as increased dyspnea, increased cough, increased sputum volume or change in sputum colour. We considered studies if >90% of patients had a clinical (physician-based) diagnosis of COPD or, ideally, spirometrically confirmed COPD. We excluded studies of patients with acute bronchitis, pneumonia, asthma or bronchiectasis.

We included trials evaluating any antibiotics that were administered orally or parenterally daily for a minimum period of at least three days. We chose three days because this is the minimum duration for which antibiotics are usually prescribed in clinical practice for COPD exacerbations.

The outcome measure of primary interest was treatment failure defined as (1) no resolution of symptoms and signs as reported by patients or physicians or as (2) need for further antibiotics. Outcome measures of secondary interest were duration of hospital admission, admission to an intensive care unit, health-related quality of life, symptoms, mortality, and any adverse events registered during the study period.

### Search strategy

The search was carried out by information specialists (Bazian, London, UK) and included searches in the Cochrane Central Register of Controlled Trials (CENTRAL, 2005 issue 4), PREMEDLINE (1960 to 1965), MEDLINE (1966 to March 2006), EMBASE (1974 to March 2006), the Database of Abstracts of Reviews of Effectiveness (DARE, March 2006). We entered all included studies into the Pub-med "related articles" function and the science citation index. In addition, we scrutinised the reference lists of included studies and review articles as well the conference proceedings of the international congresses of the American Thoracic Society and the European Respiratory Society from 2000 to 2006 since these studies might not have been fully published yet. We also contacted representatives of the pharmaceutical industry for additional published or unpublished data (Novartis, GlaxoSmithKline, AstraZeneca, BoehringerIngelheim, Pfizer and MSD). Finally, we searched international data bases for trial registration to identify ongoing or recently completed trials [14-16].

### Study selection

Two members of the review team independently assessed the titles and abstracts of all identified citations without imposing any language restrictions. The reviewers then evaluated the full text of articles that seemed potentially eligible by one of the reviewers. Final decisions on in- and exclusion were recorded in the Endnote file and agreement was assessed using chance-adjusted kappa statistics.

### Data extraction

One reviewer recorded details about study design, interventions, patients, outcome measures and results in predefined Windows Excel forms and a second reviewer checked data extraction for correctness. We used a small sample of studies with high likelihood for inclusion to pilot test the data form. To obtain missing information, we tried to contact authors of primary studies at least three times by telephone or email.

We entered dichotomous data on into 2 × 2 tables. For continuous outcomes, we recorded summary estimates per group (means, medians) with measures of variability (SD) or precision (SEM, CI). In trials with two groups receiving different antibiotics, we treated these groups as one group if the effects of the two antibiotics did not differ statistically significantly or clinically importantly.

### Quality assessment

Two reviewers independently evaluated the quality of included trials using a list of selected quality items assessing components of internal validity [17]. We recorded the initial degree of discordance between the reviewers and corrected discordant scores based on obvious errors. We resolved discordant scores based on real differences in interpretation through consensus or third party arbitration.

### Statistical analysis

We expressed treatment effects as odds ratios with corresponding 95% confidence intervals (CI) and calculated, based on pooled odds ratios, numbers-needed-to-treat. We pooled data across studies only in absence of significant heterogeneity (p > 0.1 for χ^2^) using fixed effects models (inverse variance method). We analysed comparisons with events only in one group by adding 0·5 to "zero-cells".

We assessed heterogeneity using χ^2 ^statistic and expressed the proportion of variation due to heterogeneity as I^2 ^[18]. We explored sources of heterogeneity using meta-regression following a priori defined explanations, which included severity of exacerbation (defined as severe if requiring inpatient treatment and as mild to moderate requiring outpatient treatment according to the Operational Classification of Severity of the European Respiratory and American Thoracic Societies [19]), generation of antibiotics (before and after 1980), definition of outcomes, length of follow-up (≤ and > 10 days) and study quality. We assessed publication bias using the regression-based test of Egger [20].

We conducted all analyses with STATA for windows version 8.2, Stata Corp; College Station, TX)

## Results

### Identification of studies

**Figure 1:**
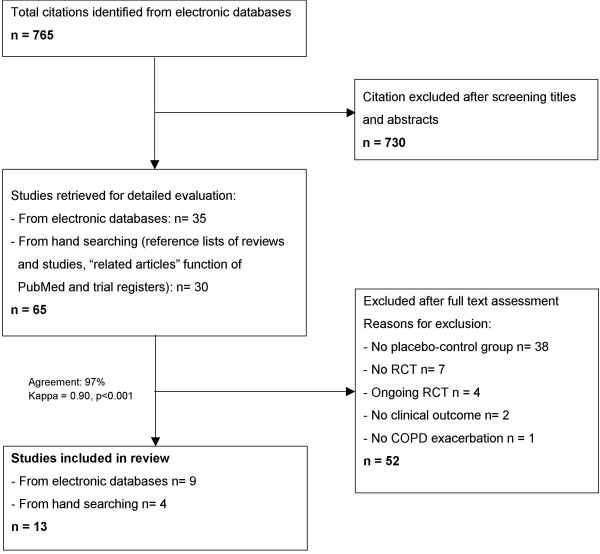
Study flow from identification to final inclusion of studies.

Figure 1 summarises the process of identifying eligible clinical trials. We identified 765 citations from electronic databases and selected 35 of them for full text assessment. Together with 30 additional citations from hand-searching we studied 65 publications in detail. We included 13 trials with 1557 COPD patients in the analyses. We excluded most trials because they compared different antibiotics without having a placebo control group. From trial registers, we identified four randomised trials that are still ongoing [21-24]. The pharmaceutical industry did not provide any unpublished data.

### Study characteristics

Table 1 shows the characteristics of the trials that were published between 1957 and 2001. In seven trials, patients suffered from mild to moderate exacerbations receiving outpatient treatment [25-31]. Six trials included patients admitted to the hospital because of severe exacerbations [32-37]. Nouira [34] included patients with very severe exacerbations, who needed mechanical ventilation. Severity of underlying COPD could not be compared across trials because lung function and other parameters were reported inconsistently between 1957 and 2001. In all trials, patients received co-interventions such as systemic corticosteroids, theophylline, β-mimetics, gastric ulcer prophylaxis or ventilation support with or without oxygen. But the proportion of patients receiving co-interventions was rarely specified and could not be considered as potential confounders in the analyses.

**Table 1:** Characteristics of included trials

**Study**	**Population**	**Interventions**	**Outcomes and length of follow-up**
Elmes 1957 [28]	88 COPD patients (84% males, mean age 54 years). Patients were instructed to take antibiotic/placebo without a doctor visit as soon as new or aggravated respiratory symptoms were present. Severity of exacerbation: Mild to moderate	**Group 1**: Oxytetracycline 1 g/day per os for 5–7 days **Group 2**: Placebo for 5–7 days	Treatment success/failure (need for further antibiotics), time off work, number of days with symptoms Mean follow-up: 17 days
Berry 1960 [27]	58 COPD patients (53% males, mean age 59 years) with general practitioner visit for new or aggravated respiratory symptoms. Patients with severe exacerbations were not included because antibiotics were deemed indispensable. Severity of exacerbation: Mild to moderate	**Group 1**: Oxytetracycline 1 g/day per os for 5 days **Group 2**: Placebo for 5 days	Treatment success/failure (patient reported) Mean follow-up: 14 days
Fear 1962 [29]	62 COPD patients (% males and mean age not stated) with outpatient visit to Bronchitis and Asthma Clinic for new or aggravated respiratory symptoms. Severity of exacerbation: Mild to moderate	**Group 1**: Oxytetracycline 1 g/day per os for 7 days **Group 2**: Placebo for 7 days	Improvement of symptoms, days of illness Mean follow-up: 14 days
Petersen 1967 [35]	19 COPD patients (53 % males, mean age 62 years) with hospital admission for exacerbation. Severity of exacerbation: Severe	**Group 1: **Chloramphenicol 2 g/day (route of administration unclear) for 10 days **Group 2: **Placebo for 10 days	Mortality, patient-reported well-being Mean follow-up: 10 days
Pines 1968 [37]	30 COPD patients (% males not stated, mean age 68 years) with hospital admission for exacerbation. Severity of exacerbation: Severe	**Groups 1**: Penicillin 6 million units and streptomycin 1 g/day parenterally for 14 days **Group 2**: Placebo for 14 days	Treatment success/failure (physician reported), mortality Mean follow-up: 14 days
Pines 1972 [36]	259 COPD patients (100% males, mean age 71 years) with hospital admission for exacerbation. Patients with very severe exacerbation were not included because antibiotics were deemed indispensable. Severity of exacerbation: Severe	**Groups 1 and 2**: Tetracycline 2 g or chloramphenicol 2 g/day per os for 12 days **Group 3**: Placebo for 12 days	Treatment success/failure (physician reported), mortality, incidence of relapses Mean follow-up: 12 days
Anthonisen 1987 [26]	116 COPD patients (80% males, mean age 67 years). Initially, 173 patients were included for observation. Of these, 116 reported worsening of respiratory symptom and received randomly assigned antibiotics or placebo on an outpatient base. 57 patients did not experience an exacerbation. Severity of exacerbation: Mild to moderate	**Group 1**: Trimethoprim-sulfamethoxazol 1.9 g or amoxicillin 1 g or doxycycline 0.1–0.2 g/day per os for 10 days **Group 2**: Placebo for 10 days	Treatment success/failure (patient reported symptoms) Follow-up: 21 days
Manresa 1987 [33]	19 COPD patients (% males not stated, mean age 67) with hospital admission for exacerbation. Severity of exacerbation: Severe	**Group 1: **Cefaclor 1.5 g/day per os for 8 days **Group 2: **Placebo for 8 days	Duration of hospitalisation Mean follow-up: 13 days
Allegra 1991 [25]	335 COPD patients (73% males, mean age 63 years). Patients received antibiotic/placebo on an outpatient base in case of self-reported worsening of respiratory symptoms. Severity of exacerbation: Mild to moderate	**Group 1**: Amoxicillin-clavulanic acid 2 g/day per os for 5 days **Group 2**: Placebo for 5 days	Treatment success/failure (patient reported symptoms and clinical signs) Mean follow-up: 5 days
Alonso Martinez 1992 [32]	90 COPD patients (84% males, mean age 68 years) with hospital admission for exacerbation. Severity of exacerbation: Severe	**Groups 1 and 2: **: Trimethoprim-sulfamethoxazol 1.9 g or amoxicillin-clavulanic acid 1.9 g/day per os for 8 days **Group 3: **Placebo for 8 days	Treatment success (need for further antibiotics), duration of hospitalisation Mean follow-up: 8 days
Jorgensen 1992 [30]	270 COPD patients (43% males, mean age 60 years) with general practitioner visit for new or aggravated respiratory symptoms. Severity of exacerbation: Mild to moderate	**Group 1**: Amoxicillin 1.5 g/day per os for 7 days **Group 2**: Placebo for 7 days	Treatment success/failure (patient reported symptoms) Mean follow-up: 8 days
Sachs 1995 [31]	61 COPD patients (% males not stated, mean age not stated) with general practitioner visit for new or aggravated respiratory symptoms. Severity of exacerbation: Mild to moderate	**Groups 1 and 2**: Amoxicillin 1.5 g or co-trimoxazol 1.9 g/day per os for 7 days **Group 3**: Placebo for 7 days	Treatment success/failure (patient reported symptoms) Mean follow-up: 35 days
Nouira 2001 [34]	93 COPD patients (90% males, mean age 66 years) with admission to intensive care unit for exacerbation and need for mechanical ventilation. Severity of exacerbation: Severe	**Group 1**: Ofloxacin 0.4 g/day per os for 10 days **Group 2**: Placebo for 10 days	Treatment success (need for further antibiotics), mortality, duration of hospitalisation Mean follow-up: 10 days

Ten trials used treatment failure as an outcome although definitions varied from patient reported failure of symptom resolution to the physicians' decision to prescribe additional treatment [25-28,30-32,34,36,37]. Four trials including patients with severe exacerbations assessed mortality [34-37] and three trials [32-34] the duration of hospital stay.

In one trial, analyses were based on the number of 116 patients with exacerbations as well as on the total number of exacerbations (n = 362) [26]. In our meta-analyses, we considered the analysis based on the number of patients only because the other trials also followed this approach. In addition, Anthonisen et al used a cross-over design for patients with more than one exacerbation. Thereby, patients with more than one exacerbation counted in the antibiotic and placebo group. In addition assessing antibiotics with a cross-over design may not fulfil the important requirement for cross-over studies that patients must return to their baseline state before starting the cross-over. COPD patients often do not fully recover from exacerbations and are, therefore, unlikely to return to their baseline state.

The quality of the trials was moderate to good (table 2). Ten trials described their method of randomisation. Concealment of random allocation was reported in eight trials and in nine trials, outcome assessors were blinded. Initial agreement for quality assessment among the two reviewers was high (88% for all items, chance-corrected kappa = 0.75, p < 0.001).

**Table 2:** Quality assessment

	Description of randomisation procedure	Pre- stratification	Concealment of random allocation	Description of loss to follow-up	Blinding of patients	Blinding of treatment providers	Description of co- interventions	Blinding of outcome assessors	Intention-to-treat-analysis	Adjustment for imbalances
Elmes 1957 [28]	1	0	1	1	1	1	1	1	1	0
Berry 1960 [27]	1	0	1	0	1	1	1	1	1	0
Fear 1962 [29]	1	0	1	1	1	1	0	1	1	0
Petersen 1967 [35]	1	1	0	1	1	0	0	0	0	1
Pines 1968 [37]	1	0	1	1	1	1	0	1	1	0
Pines 1972 [36]	1	0	1	1	1	1	1	1	1	0
Anthonisen 1987 [26]	1	0	0	1	1	1	1	1	1	0
Allegra 1991 [25]	0	0	0	1	1	1	1	1	0	0
Alonso Martinez 1992 [32]	1	0	1	1	1	1	1	0	1	0
Jorgensen 1992 [30]	0	0	0	1	1	1	1	1	1	1
Sachs 1995 [31]	1	0	1	1	1	1	1	0	1	0
Nouira 2001 [34]	1	0	1	1	1	1	1	1	1	0

0 = not addressed; 1 = partially or fully addressed

### Effects of antibiotics

Median treatment failure rate was 0.12 for the antibiotic groups (range 0.00 to 0.47) and 0.34 for the placebo groups (range 0.10 to 0.80). Thus across all trials, one out of eight patients with antibiotics had a treatment failure whereas one out of three patients had a treatment failure with placebo.

**Figure 2:**
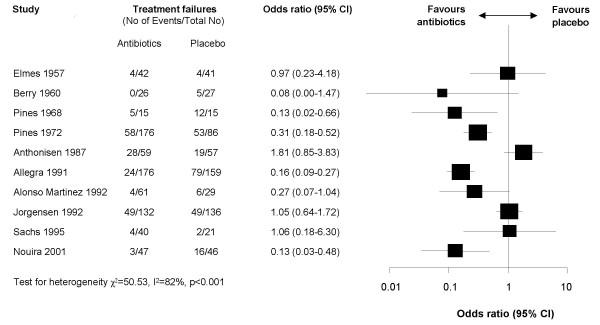
Forest plot showing ten studies that compared the effects of antibiotics and placebo on treatment failure. The x-axis represents the odds ratio for treatment failure. An odds ratio below 1 represents a lower chance of treatment failure with antibiotics. Studies not reporting treatment failures could not be included in the meta-analysis.

Figure 2 shows that the effects of antibiotics were very heterogeneous across trials (I^2 ^= 82%). When we explored predefined sources of heterogeneity in meta-regression analyses we found that generation of antibiotic (p = 0.55), definition of outcomes (p = 0.20), length of follow-up (p = 0.38) and study quality (p = 0.92) did not explain heterogeneity. We could not assess severity of COPD as a source of heterogeneity because lung function parameters were not reported in earlier trials.

Across nine of ten trials effects of antibiotics were substantially larger in patients with severe exacerbations. One trial in patients with mild to moderate exacerbations totally contradicted this trend with an unexpectedly large effect (OR 0.16, 95% 0.09–0.27) [25]. But this trial differed substantially from other trials including patients with mild to moderate exacerbations. It had a short follow-up of 5 days and a treatment failure rate of 0.50 in control patients (median follow-up of 17 days and median treatment failure rate of 0.19). After five days, adjustment of exacerbation treatment is important but seems too early to determine whether treatment was successful or not. Exacerbations last longer than five days so that effectiveness of interventions should be evaluated later on as it was the case in the other trials [38]. It must be stated that this trial actually had a follow-up assessment after 14 days but these data were not provided in the publication. In a personal communication, one of the authors told us that treatment effects were smaller at that 14 days follow-up but he was unable to provide the data because they are stored by the pharmaceutical company funding the trial [39].

**Figure 3:**
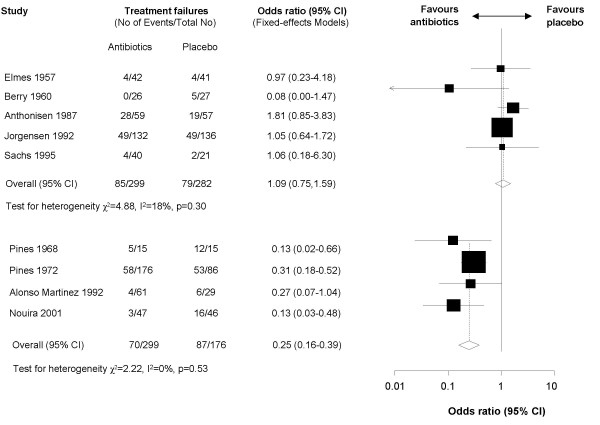
Forest plot showing nine studies grouped according to severity of exacerbation. One study with a substantially higher treatment failure rate and a short follow-up of five days was not considered in the analysis. The upper five studies included patients with mild to moderate exacerbations and the four studies below included patients with severe exacerbations. The x-axis represents the odds ratio for treatment failure. An odds ratio below 1 represents a lower chance of treatment failure with antibiotics. Studies not reporting treatment failures could not be included in the meta-analysis.

When we did the meta-analysis without this trial, we found that severity of exacerbations was associated significantly with treatment effects (p = 0.016). Figure 3 shows the pooled results separately for trials including patients with mild to moderate exacerbations and patients with severe exacerbations. For mild to moderate exacerbations, antibiotics did not significantly reduce the risk for treatment failure (OR 1.09, 95% CI 0.75–1.59, I^2 ^= 18%). When the Allegra trial [25] was included in the meta-analysis the pooled estimate favoured antibiotics (OR 0.55, 95% CI 0.41–0.74, with a number-needed to treat of 9, 95% CI 6–16) but there was a large amount of heterogeneity (I^2 ^= 87%). Antibiotics had a large effect in severe exacerbations (OR 0.25, 95% CI 0.16–0.39, I^2 ^= 0%) with a number-needed to treat of 4 (95% CI 3–5).

Effect modification by severity of exacerbation was confirmed by subgroup analyses of the trial that also presented comparisons based on exacerbations as described above [26]. There was no statistically significant effect on treatment failure rates in mild exacerbations (Anthonisen type 2 and 3 corresponding to the presence of one or two aggravated symptoms including more severe dyspnea, increased sputum volume and sputum purulence [26], OR 0.63, 95% CI 0.25–1.60) whereas in more severe exacerbations (Anthonisen type 1, presence of all three symptoms) the effect reached statistical significance (OR 0.37, 95% CI 0.16–0.85) with a number-needed to treat of 5 (95% CI 3–25).

**Figure 4:**
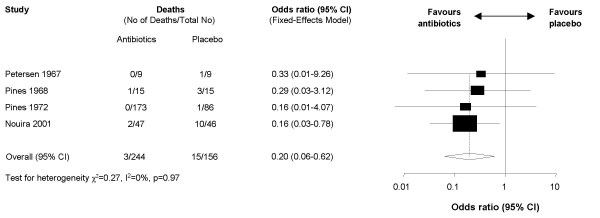
Forest plot showing the four studies that included patients with severe exacerbations. The x-axis represents the odds ratio for mortality. An odds ratio below 1 represents a lower chance of mortality with antibiotics. Studies not reporting mortality could not be included in the meta-analysis.

Effects of antibiotics on mortality confirmed the beneficial effect for patients with severe exacerbations (Figure 4). Antibiotics reduced mortality substantially (OR 0.20, 95% CI 0.06–0.62, I^2 ^= 0%) and the number needed to treat to prevent one death was 14 (95% CI 12–30).

Duration of hospital admission was not reduced in two trials (difference between groups 0.5 days, 95% CI -3.1–4.1 [33] and -0.3 days, 95% CI -1.3–0.7[32]) whereas in patients with very severe exacerbations requiring mechanical ventilation, hospital admission could be shortened by 9.6 days (95% CI 6.4–12.8) [34].

### Adverse effects

**Figure 5:**
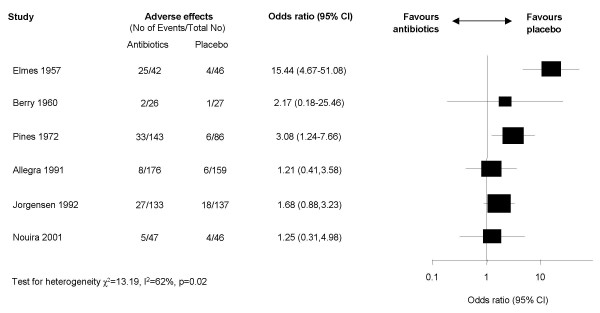
Forest plot showing six studies reporting on adverse effects. The x-axis represents the odds ratio for adverse effects. An odds ratio above 1 represents a lower chance of adverse effects with placebo. Studies not reporting adverse effects could not be included in the meta-analysis.

Median rate for adverse effects (mostly mild gastrointestinal complaints) was 0.15 (range 0.05–0.60) for the antibiotic and 0.08 (range 0.04–0.13) for the placebo groups (figure 5). In two studies, adverse effects occurred significantly more often in the placebo groups. We did not pool the results statistically because there was significant heterogeneity (I^2 ^= 62%).

### Publication bias

The Egger test of heterogeneity (regression coefficient -0.11, 95% CI -2.37–2.15, p = 0.91) did not reveal any publication bias.

## Discussion

### Principal findings

This systematic review shows that the effects of antibiotics are likely to depend on the severity of COPD exacerbations. The meta-analyses indicate that COPD patients with mild to moderate exacerbations may not benefit from antibiotics as part of the exacerbation treatment. In contrast, trials including patients with severe exacerbations showed that antibiotics led to a substantial reduction in treatment failure and mortality rates.

### Strengths and weaknesses

Strengths of this study include adherence to rigorous systematic review methodology, the comprehensive literature search and contacts to authors who provided additional information [25,31]. Furthermore, we carefully addressed heterogeneity of study results using predefined, clinically plausible sources of heterogeneity in formal meta-regression analysis.

Although treatment failure is commonly used in meta-analyses [12,40], it is a limitation that definitions of treatment failure often differ across trials. It is difficult to standardise the definition of treatment failure because it may include patient reported symptoms, clinical signs and results from laboratory tests or imaging. We do not, however, have reason to believe that different definitions of treatment failure caused heterogeneity in our meta-analyses. Another limitation is that severity of underlying COPD could not be studied as potential source of heterogeneity. The definitions and classifications of COPD changed over the years so that no uniform classifications of COPD such as the GOLD stages could be extracted from the studies. Also, we could not assess the influence of other factors such as season, co-morbidities or co-medications such as systemic steroids or bronchodilators as they were reported poorly and inconsistently. Finally, the included trials did not study patient-important outcomes such as health-related quality of life, which is heavily influenced by exacerbations [41] and one of the main targets of COPD treatments [19].

### Meaning of the study

We quantified the influence of severity of exacerbations on the effects of antibiotics using the Operational Classification of Severity of the European Respiratory and American Thoracic Society [19]. The major advantage of this classification over earlier ones [26] is that it is simple to apply. But one needs to consider that severity of exacerbations is not the only determinant for hospital admission and that co-morbidity and social circumstances also play an important role. As long as the mechanisms of exacerbations are not fully understood and cannot be assessed in detail by pathophysiological variables, the Operational Classification of Severity may describe exacerbations most comprehensively. This simplification comes at the price of not discriminating between different forms of exacerbations that can be treated on an outpatient base. It is, however, unclear, whether this distinction is necessary in general. Even if there is an effect of antibiotics in more severe exacerbations of outpatients it is likely to be small. The four ongoing trials, that all include outpatients, may inform us in this regard [21-24].

The results of our systematic review may have important implications for clinical practice and help to inform discussions that are ongoing for decades. Most patients with COPD exacerbations who do not need hospital admission may not benefit from immediate antibiotic treatment. The most prudent choice for these patients might be to withhold antibiotics at first while first line management should include bronchodilators, systemic corticosteroids, patient instruction to use medications correctly as well as follow-up visits [3,19]. If patients do not recover or show further worsening of health status, antibiotics might still be considered after 3 to 5 days of first line treatment. Thereby, a substantial amount of antibiotics could be spared with positive consequences for the patient and society (adverse effects, antibiotic resistance and costs.)

### Unanswered questions and future research

To base this proposed strategy on solid grounds, a randomised, non-inferiority trial comparing the clinical effectiveness and amount of antibiotics used with immediate antibiotic treatment and a watchful-waiting strategy would be highly welcome. Thereby, investigators could show whether a watchful-waiting strategy is clinically not disadvantageous but associated with reduced use of antibiotics.

Factors other than treatment setting that may guide antibiotic treatment also deserve further research. For example, studies showed promising results for procalcitonin guidance of antibiotic treatment in lower respiratory tract infections and might be evaluated for COPD exacerbations as well [42, 43]. Also, a recent study showed that patient-reported sputum purulence was an excellent predictor of positive bacteria cultures [44]. Although the study was too small for multivariable analyses and no patient-important outcomes were assessed, the usefulness of sputum purulence to guide antibiotic treatment should be further studied.

Finally, future studies should explore the long-term effects of antibiotics when given for acute exacerbations. The trials included in this review only assessed the effects on short-term outcomes such as treatment failure or mortality. However, it may be possible that antibiotics eradicate bacteria that could cause exacerbations in the future. Thus antibiotics might prolong the exacerbation-free interval or even reduce the number of exacerbations.

## Conclusion

Our systematic review informs the debate about appropriate prescription of antibiotics for COPD exacerbations. As long as exacerbations remain an ill-defined event, the distinction between in- and outpatient treatment may serve as simple guidance to decide for or against antibiotics. Patients with severe exacerbations requiring hospital admission benefit substantially from antibiotics. In outpatients with mild to moderate exacerbations, antibiotics appear to offer no benefits in general. Further research will show how the subgroup of patients with mild to moderate exacerbations, who might benefit from antibiotics, can be identified.

## Authors' contributions

MP participated in the design of the study, checked the data, performed the statistical analysis and drafted the manuscript. DV collected the data and revised the manuscript. TL collected the data. JS participated in the design of the study and revised the manuscript. CS participated in the design of the study and revised the manuscript. All authors read and approved the final manuscript.

## Conflict of interest statement

The author(s) declare that they have no competing interests.

## Funding

The Lung League of Zurich funded this study with an unrestricted grant. Milo Puhan is supported by a career award of the Swiss National Science Foundation (grant # 3233B0/115216/1). The sponsors had no role in study design, data collection, data analysis, data interpretation, or writing of the report. The corresponding author had full access to all the data in the study and had final responsibility for the decision to submit for publication.
