# Exacerbations of chronic obstructive pulmonary disease: When are antibiotics indicated? A systematic review

**DOI:** 10.1186/1465-9921-9-81

**Published:** 2008-12-15

**Authors:** Milo A Puhan, Daniela Vollenweider, Tsogyal Latshang, Johann Steurer, Claudia Steurer-Stey

**Affiliations:** 1 Horten Centre, University Hospital of Zurich, Postfach Nord, CH-8091 Zurich, Switzerland

## Correction

Since publication of our article [1], we have been made aware of an error in our article.

We have transposed the proportion of patients with treatment failure in the antibiotic and placebo groups of one trial [2]. 19 out of 57 patients experienced a treatment failure compared to 28 out of 59 patients in the placebo group (odds ratio 0.55 (0.26–1.17).

In the results section of the abstract the sentences "For the effects of antibiotics on treatment failure there was much heterogeneity across all trials (I^2 ^= 82%). Meta-regression revealed severity of exacerbation as significant explanation for this heterogeneity (p = 0.016): Antibiotics did not reduce treatment failures in outpatients with mild to moderate exacerbations (pooled odds ratio 1.09, 95% CI 0.75–1.59, I^2 ^= 18%)" the text should read: "For the effects of antibiotics on treatment failure there was much heterogeneity across all trials (I^2 ^= 75%). Meta-regression revealed severity of exacerbation as significant explanation for this heterogeneity (p = 0.038): Antibiotics did not reduce treatment failures in outpatients with mild to moderate exacerbations (pooled odds ratio 0.81, 95% CI 0.55–1.18, I^2 ^= 13%)."

In the results section, following the subheading *"Effects of antibiotics" *the second paragraph "Figure 2 shows that the effects of antibiotics were very heterogeneous across trials (I^2 ^= 82%). When we explored predefined sources of heterogeneity in meta-regression analyses we found that generation of antibiotic (p = 0.55), definition of outcomes (p = 0.20), length of follow-up (p = 0.38) and study quality (p = 0.92) did not explain heterogeneity." should read "Figure 2 shows that the effects of antibiotics were very heterogeneous across trials (I^2 ^= 75%). When we explored predefined sources of heterogeneity in meta-regression analyses we found that generation of antibiotic (p = 0.59), definition of outcomes (p = 0.06), length of follow-up (p = 0.85) and study quality (p = 0.42) did not explain heterogeneity."

**Figure 1:**
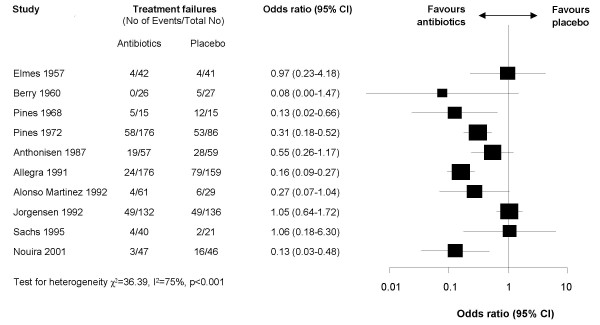
**Forest plot showing ten studies that compared the effects of antibiotics and placebo on treatment failure. The x-axis represents the odds ratio for treatment failure**. An odds ratio below 1 represents a lower chance of treatment failure with antibiotics. Studies not reporting treatment failures could not be included in the meta-analysis.

**Figure 2:**
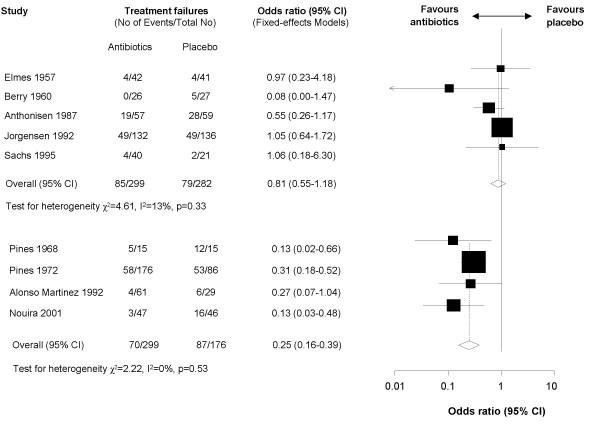
**Forest plot showing nine studies grouped according to severity of exacerbation**. One study with a substantially higher treatment failure rate and a short follow-up of five days was not considered in the analysis. The upper five studies included patients with mild to moderate exacerbations and the four studies below included patients with severe exacerbations. The x-axis represents the odds ratio for treatment failure. An odds ratio below 1 represents a lower chance of treatment failure with antibiotics. Studies not reporting treatment failures could not be included in the meta-analysis.

In the results section, following the subheading *"Effects of antibiotics" *the fourth paragraph "When we did the meta-analysis without this trial, we found that severity of exacerbations was associated significantly with treatment effects (p = 0.016). Figure three shows the pooled results separately for trials including patients with mild to moderate exacerbations and patients with severe exacerbations. For mild to moderate exacerbations, antibiotics did not significantly reduce the risk for treatment failure (OR 1.09, 95% CI 0.75–1.59, I^2 ^= 18%). When the Allegra trial [25] was included in the meta-analysis the pooled estimate favoured antibiotics (OR 0.55, 95% CI 0.41–0.74, with a number-needed to treat of 9, 95% CI 6–16) but there was a large amount of heterogeneity (I^2 ^= 87%)." should read "When we did the meta-analysis without this trial, we found that severity of exacerbations was associated significantly with treatment effects (p = 0.038). Figure three shows the pooled results separately for trials including patients with mild to moderate exacerbations and patients with severe exacerbations. For mild to moderate exacerbations, antibiotics did not significantly reduce the risk for treatment failure (OR 0.81, 95% CI 0.55–1.18, I^2 ^= 1 three%). When the Allegra trial [25] was included in the meta-analysis the pooled estimate favoured antibiotics (OR 0.45, 95% CI 0.34–0.61, with a number-needed to treat of 9, 95% CI 7–12) but there was a large amount of heterogeneity (I^2 ^= 83%)."

The corrected versions of Figures two and three are given here- see figures 1 and 2.

We apologize for any inconvenience or confusion that this may have caused.
